# Focal limb dystonia caused by a complication of the cerebellar developmental venous anomaly: a case report

**DOI:** 10.1186/s12883-019-1446-8

**Published:** 2019-09-03

**Authors:** Su Jin Chung

**Affiliations:** Department of Neurology, Myongji Hospital, Hanyang University College of Medicine, 55, Hwasu-ro 14beon-gil, Deogyang-gu, Goyang, 10475 South Korea

**Keywords:** Dystonia, Cerebellum, Dentate nucleus, Developmental venous anomaly

## Abstract

**Background:**

There are no established theories regarding the role of the cerebellum in dystonia. We report a case of focal limb dystonia secondary to a vasogenic edema of the dentate nucleus caused by a symptomatic developmental venous anomaly.

**Case presentation:**

A 44-year-old woman presented with sudden onset dystonia in her left arm for 1 week. Brain imaging revealed vasogenic edema in the deep white matter of the left cerebellar hemisphere, including the left dentate nucleus, secondary to a developmental venous anomaly. ^18^F-fluorodeoxyglucose positron emission tomography images showed hypometabolism in the corresponding cerebellar deep nuclei without the involvement of other brain regions. She was treated with a steroid. At the one-month follow-up, computed tomography scan demonstrated remission of the cerebellar edema, which was thought to be the cause of dystonia.

**Conclusions:**

This case demonstrates that the cerebellum has an important role in the development of dystonia. Further studies are needed to elucidate the relationship between dystonia and cerebellar dysfunction.

**Electronic supplementary material:**

The online version of this article (10.1186/s12883-019-1446-8) contains supplementary material, which is available to authorized users.

## Background

Dystonia is a hyperkinetic movement disorder defined by sustained or intermittent muscle contractions causing abnormal twisting, repetitive or patterned movements, and abnormal postures [1]. Dystonia has long been considered as a disorder of the basal ganglia signaling, but emerging evidence suggests that dystonia is a motor network disorder involving multiple brain regions, including the cerebellum [2]. However, the mechanisms of brain dysfunction in dystonia have not been fully investigated. Moreover, there are much fewer case reports of the cerebellar focal lesions in focal dystonia compared to that of focal lesions in the basal ganglia. We report a case of focal limb dystonia secondary to a vasogenic edema of the dentate nucleus caused by a symptomatic developmental venous anomaly (DVA).

## Case presentation

**Fig. 1:**
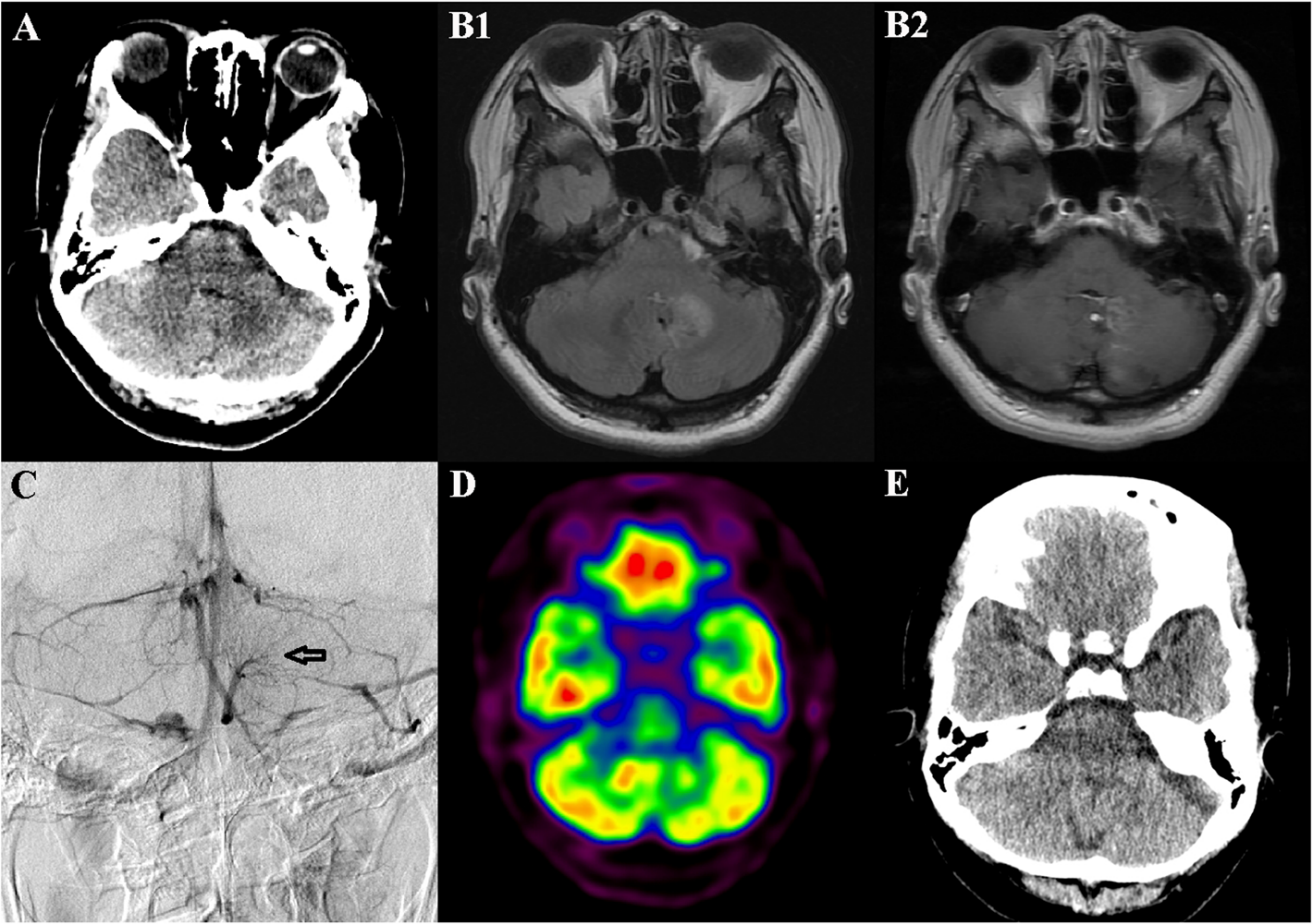
Brain imaging results. **a**: A non-contrast computed tomography (CT) image shows a hypodense lesion in the left cerebellar deep nuclei. **b**: A fluid-attenuated inversion recovery image shows vasogenic edema within the deep white matter of the left cerebellum, especially affecting the left dentate nucleus (**b1**). These signal abnormalities are observed in multiple enhancing medullary veins with caput medusa appearance in a gadolinium-enhanced T1-weighted axial image (**b2**). **c**: Venous phase of the left vertebral artery digital subtraction angiography in anteroposterior view demonstrates umbrella-shaped collections of dilated medullary veins in the left cerebellum (arrow). **d**: Asymmetrically reduced glucose metabolism in the affected left cerebellum is revealed in ^18^F-fluorodeoxyglucose positron emission tomography. **e**: A follow-up brain CT scan 1 month later shows resolution of the cerebellar edema


A 44-year-old female patient was admitted at our hospital with sudden onset of involuntary movements in her left arm for 1 week. She had no medical history. The patient showed dystonic posturing of the left hand with downward wrist flexion and hyperextension of the fingers when she outstretched both arms. Further, the left hand showed intermittent repetitive and patterned flexion of the fingers on maintaining posture. Sustained muscle contractions of the left arm also interfered with voluntary actions. In addition, she exhibited dysmetria and intention tremor of the affected arm in the finger-to-nose test (See Additional file 1). However, she had no problems in the other body parts. Other neurologic deficits, including parkinsonism or myoclonus, were not detected. Laboratory results, cerebrospinal fluid findings, and electroencephalogram recordings were all within normal limits. Non-contrast computed tomography (CT) of the brain showed low density in the left cerebellar deep nuclei (Fig. 1a). Magnetic resonance imaging (MRI) revealed vasogenic edema in the deep white matter of the left cerebellar hemisphere, including the left dentate nucleus, secondary to a DVA (Fig. 1b). Other than the DVA, no vascular malformations were found on digital subtraction angiography (Fig. 1c). The cerebellar lesion was not substantial enough to explain the pathophysiology of the patient’s dystonia; therefore, ^18^F-fluorodeoxyglucose positron emission tomography (FDG-PET) was performed to evaluate cerebral glucose metabolism within the basal ganglia and connected motor cortex. However, FDG-PET images revealed no abnormalities in other brain regions, except for hypometabolism in the corresponding cerebellar deep nuclei (Fig. 1d). Administration of intravenous methylprednisolone (1 g/day for 5 days), and then one-week tapering course of oral prednisolone (initiated at 60 mg/day) completely alleviated the symptoms after 1 month. On follow-up CT, cerebellar edema that may have been responsible for the dystonia was not detected (Fig. 1e).

**Additional file 1:** Video of the patient. Intermittent patterned flexion of the fingers of the patient’s left hand with dystonic posturing, when she was asked to keep her arms outstretched, is shown in this video. She has a problem with the voluntary actions of the left arm due to sustained muscle contractions. Additionally, dysmetria and intention tremor can be seen in the affected arm on the finger-to-nose test. (MP4 18747 kb)


## Discussion and conclusions

We report a case of a patient with acute onset of position-specific hyperkinetic movements, combined with the sustained abnormality of posture in the left hand with cerebellar signs in the left arm. The rapid hyperkinetic movements of the patient are comparable with dystonia, which is characterized by repetitive, stereotyped, and predictable movements during posture maintenance. To date, there are a few case reports of upper limb dystonia associated with cerebellar mass or atrophy [3, 4]. However, to the best of our knowledge, there are no previous reports on focal limb dystonia secondary to cerebellar edema related to a venous malformation.

Our present finding is unique in that idiopathic venous congestion occurred due to an infratentorial DVA, which, in turn, caused cerebellar dysfunction and hypometabolism that ultimately led to dystonia. In rare cases, DVAs can be symptomatic due to mechanical or flow-related pathomechanisms. Whereas mechanical complications lead either to hydrocephalus or to nerve compression syndromes, flow-related mechanisms can be subdivided into those that are related to an increased inflow into the DVA or to an obstruction of the outflow. Patients in whom neither pathomechanisms are classified as idiopathic or spontaneous symptomatology [5]. Our case belongs to idiopathic complication with no associated vascular condition nor systemic factor.

The potential role of the cerebellum in dystonia has been reported; however, the pathophysiology is still not fully understood. Recent structural and functional imaging studies suggest that the cerebellum has a role in primary dystonia [6]. A number of secondary cervical dystonia cases are associated with cerebellar and brainstem lesions rather than basal ganglia lesions [7]. In addition, dystonia can be the remarkable feature of some inherited cerebellar ataxias, although these are multisystemic diseases with degeneration not just confined to the cerebellum [8].

In the past decade, findings from animal experiments have indicated that the basal ganglia and cerebellum are interconnected at the subcortical level [9, 10]. Studies on monkeys have revealed a direct disynaptic projection from the dentate nucleus to the striatum via the intralaminar thalamic nuclei [9]. Furthermore, growing evidence suggests that the cerebellum and cerebello-basal ganglia interactions play a crucial role in the development of dystonia. In a mouse model of rapid-onset dystonia-parkinsonism, abnormal cerebellar output derived high-frequency bursting activity in the dorsolateral striatum, which receives the input from the disynaptic pathway connecting the cerebellum to the basal ganglia, led to dystonia. This cerebellar-induced dystonia was abolished by eliminating the link between the cerebellum and the basal ganglia, suggesting that the cerebellum is a critical node in the pathway leading to the development of dystonia [10]. In our case, an aberrant cerebellar output from the lesion of the dentate nucleus may modulate the activity of the basal ganglia to cause dystonia.

In recent years, imaging studies in human dystonia patients have demonstrated changes in resting cerebellar metabolism. Using diffusion tensor MRI in subjects carrying the DYT1 and DYT6 mutations, it was observed that reduction in cerebellothalamic connectivity led to increased motor activation responses, finally resulting in the loss of inhibition at the cortical level [11]. The role of the cerebellum in dystonia is also proved by the cerebellar interventions for the therapeutic approach of various dystonic syndromes. A recent study showed that bilateral deep anterior cerebellar stimulation in patients with secondary dystonia can reduce dystonic symptoms [12].

Despite imaging and neurophysiological studies in humans supporting a cerebellar origin for dystonia, it is still not clear whether these cerebellar abnormalities are causal, contributory, or compensatory. In addition, there is a lack of evidence to support the claims that the cerebellum alone can induce dystonia [2]. In one family autopsy study, the patients with generalized dystonia showed cerebello-olivary degeneration with no abnormalities in the basal ganglia and responded to deep brain stimulation of the globus pallidus internus. Eventually, it was found that dystonia was triggered by a functional failure of the basal ganglia which can be induced by abnormal signals from the cerebellum, implicating that dystonia is a basal ganglia disorder [13]. Therefore, it seems reasonable that dysfunction of the basal ganglia, cerebellum, or their connections through the thalamus or directly with the motor or premotor cortex may cause dystonia [2]. Taken together, our case has significant implications for the evidence which states that cerebellum is the key site of developing dystonia without dysfunction of the basal ganglia.

To sum up, our case report highlight several important findings. First, only dentate nucleus lesion apart from the other cerebellar areas is the trigger point of the cerebellar process cascade causing dystonia. This is consistent with the previous studies which suggest that the origin of dystonia in the cerebellar route is the dentate nucleus. Second, cerebellar edema without involving other brain structures demonstrates that cerebellum alone may result in dystonia. This confirms that the cerebellum plays a causative role, and not a compensatory role, in the development of dystonia. Third, our patient’s semiautomatic, patterned, but speedy finger flexion is very meaningful dystonia phenomenology because it is commonly much slower than that in chorea and myoclonus despite being defined as a hyperkinetic movement disorder.

In conclusion, although there are various studies on animal, imaging, neurobiology, and neurophysiology, case reports on human focal limb dystonia due to cerebellar damage are rare. The present case is sufficient to conclude that the cerebellum plays a key role in developing dystonia. Future clinical, neuroimaging, and electrophysiologic studies are needed to elucidate the relationship between dystonia and cerebellar dysfunction.

## Data Availability

All data and material (Additional file 1: video) supporting our findings are contained within the manuscript.

## Abbreviations

CT: Computed tomography
DVA: Developmental venous anomaly
FDG-PET: ^18^F-fluorodeoxyglucose positron emission tomography
MRI: Magnetic resonance imaging
